# SARS-CoV-2 Infects the Brain Choroid Plexus and Disrupts the Blood-CSF Barrier in Human Brain Organoids

**DOI:** 10.1016/j.stem.2020.10.001

**Published:** 2020-12-03

**Authors:** Laura Pellegrini, Anna Albecka, Donna L. Mallery, Max J. Kellner, David Paul, Andrew P. Carter, Leo C. James, Madeline A. Lancaster

**Affiliations:** 1 MRC Laboratory of Molecular Biology, Cambridge Biomedical Campus, Francis Crick Avenue, Cambridge CB2 0QH, UK

**Keywords:** COVID-19, SARS-CoV-2, cerebral organoids, choroid plexus organoids, blood-CSF-barrier, apolipoprotein

## Abstract

Coronavirus disease 2019 (COVID-19), caused by the severe acute respiratory syndrome coronavirus 2 (SARS-CoV-2) virus, leads to respiratory symptoms that can be fatal. However, neurological symptoms have also been observed in some patients. The cause of these complications is currently unknown. Here, we use human-pluripotent-stem-cell-derived brain organoids to examine SARS-CoV-2 neurotropism. We find expression of viral receptor ACE2 in mature choroid plexus cells expressing abundant lipoproteins, but not in neurons or other cell types. We challenge organoids with SARS-CoV-2 spike pseudovirus and live virus to demonstrate viral tropism for choroid plexus epithelial cells but little to no infection of neurons or glia. We find that infected cells are apolipoprotein- and ACE2-expressing cells of the choroid plexus epithelial barrier. Finally, we show that infection with SARS-CoV-2 damages the choroid plexus epithelium, leading to leakage across this important barrier that normally prevents entry of pathogens, immune cells, and cytokines into cerebrospinal fluid and the brain.

## Highlights

• SARS-CoV-2 entry factors are expressed in choroid plexus (ChP) cells
• More mature lipid-producing ChP cells could be more susceptible to SARS-CoV-2 infection
• SARS-CoV-2 productively infects ChP, but not neurons, in organoids
• SARS-CoV-2 infection damages the ChP epithelium, causing leakage of this brain barrier

## Graphical Abstract

**Figure:**
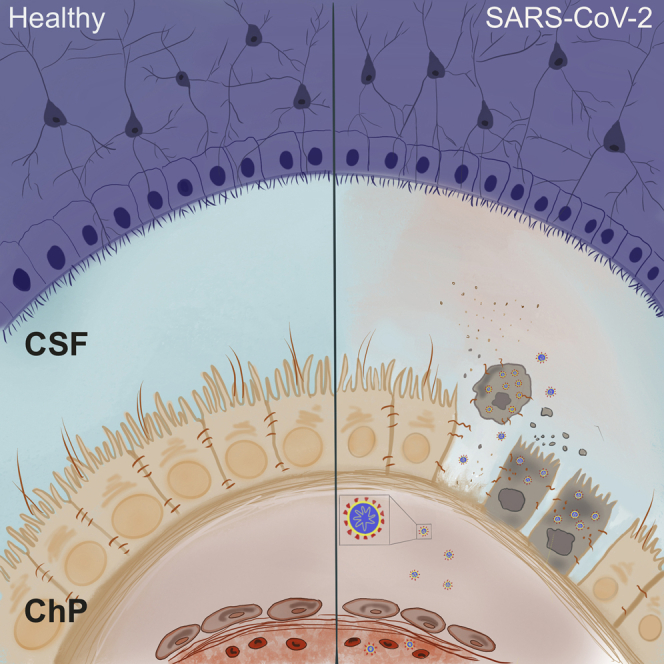

## Teaser

Increasing reports show neurological symptoms in COVID-19 patients, but it is still unclear whether SARS-CoV-2 can directly damage neurons. Lancaster and colleagues investigate viral neurotropism using brain organoids and discover that SARS-CoV-2 does not significantly infect neurons but productively infects choroid plexus, leading to damage of this brain barrier.

## Introduction

The coronavirus disease 2019 (COVID-19) global pandemic caused by the severe acute respiratory syndrome coronavirus 2 (SARS-CoV-2) has infected more than 32 million people and caused around 990,000 deaths as of late September 2020 (https://worldometers.info/coronavirus), and it still poses a constant threat to public health systems worldwide. Respiratory symptoms, predominantly associated with the infection, include fever, chest tightness, and persistent cough (Wu et al., 2020).

SARS-CoV-2 shares around 80% sequence similarity with SARS-CoV (Wang et al., 2020), and both use their spike (S) glycoprotein, composed of two subunits S1 and S2, to mediate host cell entry (Wang et al., 2020; Wu et al., 2020). S1 facilitates viral attachment to a cell surface receptor, angiotensin-converting enzyme 2 (ACE2), and S2 is essential for membrane fusion (Hoffmann et al., 2020b, 2020a). Co-entry factors, such as TMPRSS2 (Hoffmann et al., 2020a), TMPRSS4 (Zang et al., 2020), and neuropilin1 (Cantuti-Castelvetri et al., 2020; Daly et al., 2020), have also been reported to potentiate infectivity.

Even though respiratory symptoms seem to be the most prominent, increasing evidence from clinical reports indicates a rise in both acute and chronic neurological symptoms, including headache, seizures, confusion, and even psychosis (Varatharaj et al., 2020), as well as long-term complications, such as persistent fatigue (Montalvan et al., 2020) and meningitis/encephalitis (Moriguchi et al., 2020). These symptoms may indicate viral tropism for brain cells, or they may be due to more indirect effects as a result of systemic inflammation. Interestingly, one case reported SARS-CoV-2 in the cerebrospinal fluid (CSF) (Moriguchi et al., 2020), and additional autopsy findings have demonstrated viral presence within the brains of some patients (Song et al., 2020). However, the prevalence of central nervous system (CNS) infection is not yet known, and viral presence in the brain and CSF is not a widely reported finding (Helms et al., 2020; Neumann et al., 2020; Schuler et al., 2020).

The CNS is protected from the rest of the body by two major barriers: the blood-brain barrier (BBB) and the blood-CSF barrier (B-CSF-B). Both barriers prevent entry of blood-borne toxins and pathogens, including viruses. Thus, these barriers represent a major obstacle to SARS-CoV-2 infection of the brain, and it is not yet clear how the virus may enter the brain. Because tropism for nasal epithelial cells has been demonstrated (Sungnak et al., 2020), viral entry from the nasal cavity through the cribriform plate into the olfactory bulb has been suggested as a potential route of entry to the CNS (Montalvan et al., 2020). However, *post mortem* autopsies suggest this route is unlikely in humans (Schuler et al., 2020).

The BBB, which separates the systemic blood from the brain parenchyma, is a complex barrier constituted by multiple cell types and mainly formed by the tight junctions between endothelial cells along with pericytes and glial endfeet. It therefore represents a complex and highly insulated barrier. The B-CSF-B instead is much simpler, being formed by a single layer of epithelial cells of the choroid plexus (ChP) that separate the fenestrated, leaky capillaries of the stroma from the CSF (Ghersi-Egea et al., 2018; Lehtinen et al., 2011; Lun et al., 2015; Strazielle and Ghersi-Egea, 2013). The stroma is a rich environment that also provides a site of immune surveillance, as well as acting as a gateway for immune cells (Schwerk et al., 2015). This close interaction with the blood and immune cells makes the ChP epithelium particularly exposed, and previous studies have suggested its invasion may be responsible for the encephalitis caused by lentivirus and the virus Coxsackievirus B3 (CVB3) (Schwerk et al., 2015). In addition, the ChP itself may contribute to the immune response of the host by secreting proinflammatory cytokines, such as interleukin-6 (IL-6) and IL-8, into the CSF (Schwerk et al., 2015).

Because human brain tissue is difficult to access, particularly from patients with a contagious pathogen due to safety concerns (Hanley et al., 2020), 3D *in vitro* models, called cerebral organoids, can provide a viable and safe alternative. These tissues can faithfully recapitulate various aspects of human neuronal organization and function (Giandomenico et al., 2019; Lancaster et al., 2017). Indeed, several published and preliminary reports have used neural organoids to demonstrate some degree of neurotropism (Ramani et al., 2020; Song et al., 2020). However, the physiological relevance is still unclear, in particular, the degree of infection relative to more susceptible cell types as well as the route of entry into the brain.

We recently developed an organoid model to study the ChP (Pellegrini et al., 2020), which recapitulates the epithelial polarization of ChP cells and the formation of a tight barrier that separates the surrounding media from the CSF-like fluid secreted by the ChP. To test viral tropism of SARS-CoV-2 in various cells of the CNS, we examined the expression patterns of viral entry factors in cerebral and ChP organoids and tested for infection with both pseudovirions carrying SARS-CoV-2 spike and live SARS-CoV-2. We found that particular lipoprotein-producing cells of the ChP expressed SARS-CoV-2 entry factors. Comparison with *in vivo* data supported these findings and suggested these cells represent highly mature ChP epithelial cells. We then tested infection with SARS-CoV-2 spike pseudovirions and live virus, which could productively infect ChP epithelial cells. In contrast, neurons and other CNS cell types were not generally susceptible, except under infection with very large viral quantities. Finally, we observed that the primary effect of the virus was on ChP cells, which disrupted integrity of this key CNS barrier and caused it to become leakier.

## Results

### ACE2 and Other Entry Factors Are Expressed in the Choroid Plexus

**Figure 1:**
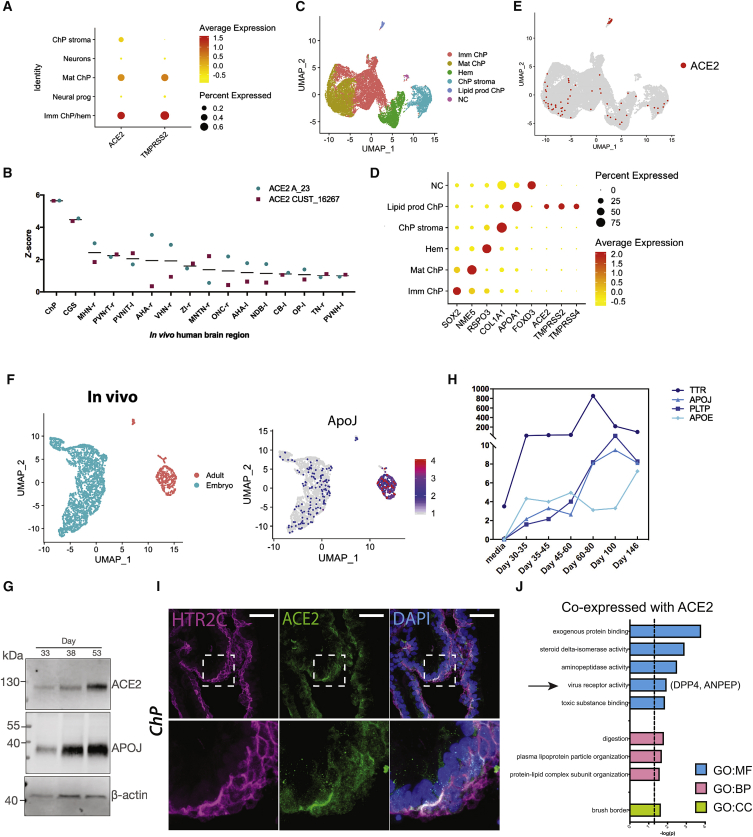
ACE2 and Other Entry Factors Are Expressed in the Choroid Plexus (A) Dot plot showing average expression and percentage of cells expressing SARS-CoV-2 entry factors ACE2 and TMRPSS2 in the five main clusters identified by scRNA-seq of days 27–53 ChP and day 55 telencephalic organoids (Pellegrini et al., 2020). (B) Allen Brain Atlas expression data of ACE2 (probe names: ACE2 A_23 and ACE2 CUST_16267) in different adult human brain regions, with highest expression in the ChP. Regions with an average *Z* score across the two probes (black line) of greater than 1 are shown. (C) Uniform Manifold Approximation and Projection (UMAP) plot showing subclustering of all ChP cell types identified by scRNA-seq. Imm ChP, immature ChP; lipid prod ChP, lipoprotein-producing ChP; Mat ChP, maturing ChP; NC, neural crest. (D) Dot plot showing average expression and percentage of cells for key marker genes present in the subclusters identified by scRNA-seq. Lipoprotein-producing ChPs express SARS-CoV-2 entry genes ACE2, TMPRSS2, and TMPRSS4. (E) Feature plot showing all cells expressing any level of ACE2. (F) UMAP plot of mouse embryonic and adult ChP cells (left) and feature plot for ApoJ (clusterin). (G) Immunoblot for ACE2, APOJ, and the loading control β-actin of days 33–53 ChP organoid protein lysates. (H) Exponentially Modified Protein Abundance Index (emPAI) values for lipid-related proteins in a previously published dataset (Pellegrini et al., 2020) of organoid CSF samples until day 146. (I) Representative confocal images of day 40 ChP tissue immunostained for the ChP epithelial marker HTR2C (serotonin receptor 2C) in magenta and ACE2 in green. Nuclei in blue are stained with DAPI. Scale bar: 50 μm. (J) gProfileR (Reimand et al., 2011) analysis of genes co-expressed with ACE2 showing significant enrichment (p < 0.05) for GO categories cellular component (GO:CC), molecular function (GO:MF), and biological process (GO:BP).

To assess whether SARS-CoV-2 entry factors are present in various cell types in brain organoids, we looked at the expression of the receptor ACE2 and the co-entry factor TMPRSS2 in different clusters of cells from previously published single-cell RNA sequencing (scRNA-seq) data from ChP and telencephalic organoids (Pellegrini et al., 2020; Figure 1A). Expression of ACE2 and TMPRSS2 was detected predominantly in ChP clusters, but not in the neural progenitor or neuron clusters (Figure 1A). To examine whether these results were in agreement with the expression *in vivo*, we analyzed data from the Allen Brain Atlas (Hawrylycz et al., 2012; Miller et al., 2017) reporting expression levels of ACE2 in different human brain regions (Figure 1B). Among all the different brain regions compared, we found highest levels of ACE2 in the ChP (Figure 1B), validating our findings *in vitro*.

To better characterize the distribution of SARS-CoV-2 entry factors in the different ChP populations, we performed subclustering of all the ChP cell populations, and we identified four prominent clusters of immature ChP, maturing ChP, hem and ChP stroma, as well as two smaller clusters: one that appeared to be neural crest cells and one that was enriched in expression of lipoprotein genes, such as APOA1 and APOA2 (Besler et al., 2012; Wang et al., 2019), suggesting a function in lipid production and transfer (Figures 1C, 1D, and S1A). We then looked at the main SARS-CoV-2 entry genes in these cell populations and found the highest expression of ACE2 in lipoprotein-expressing ChP cells (Figures 1D, 1E, and S1B) although some cells of other ChP clusters expressed ACE2 to a lower extent (Figures 1E and S1B). Similarly, the co-entry factors TMPRSS2 and TMPRSS4 were most abundant in ChP cells with high levels of lipoprotein expression (Figures 1D and S1B).

The presence of a cluster of ChP cells expressing abundant lipoproteins was intriguing. In order to examine whether such lipoprotein-producing cells were similarly present *in vivo*, we examined available scRNA-seq datasets from embryonic and adult mouse (Cao et al., 2019; Zeisel et al., 2018). We extracted those cells specifically expressing ChP markers and performed cluster analysis. This revealed clustering mainly by age, with embryonic and adult cells confined to separate clusters (Figure 1F). Examination of apolipoproteins revealed their enrichment in the major adult ChP cluster, which exhibited much higher levels than cells of the embryonic ChP (Figures 1F and S1C). This suggests that the lipoprotein-producing cells of ChP organoids may represent a more-mature ChP stage.

To explore this timing further, and to examine whether protein levels displayed a similar pattern, we performed immunoblotting for both ACE2 and APOJ, an abundant CSF lipoprotein present in various-sized lipoprotein particles (Wang and Eckel, 2014). We could detect both APOJ and ACE2 in ChP organoid lysates with increasing abundance over time (Figure 1G). Furthermore, we examined a previously published proteomic dataset of CSF-like fluid from ChP organoids spanning day 32 to day 146 (Pellegrini et al., 2020), which revealed increasing secretion of lipoproteins with age (Figure 1H). These findings further point to lipoprotein production as a hallmark of maturity and suggest that ACE2 expression is upregulated in these more mature cells.

Next, to validate the observed differences in expression of ACE2 in neuronal and ChP cells, we performed immunostaining of organoids containing cortical (Figure S1D) or ChP identities (Figure 1I). Consistent with scRNA-seq data, the ChP epithelial tissue, marked by the serotonin receptor HTR2C, showed sparse but strong positive signal for ACE2 compared to cortex. Together, these findings indicate that ACE2 is expressed in cells of the ChP, but no specific expression of ACE2 is present in neuronal progenitors or neurons. Expression of TMPRSS2 was also detected by immunostaining in the membrane of ChP epithelial cells from organoids (Figure S1E).

To further explore the expression profile of these identified ACE2-expressing ChP cells, we investigated which genes showed correlated expression pattern with ACE2. Gene Ontology (GO) analysis of genes co-expressed with ACE2 revealed enrichment in GO:molecular function (MF) categories “exogenous protein binding,” “viral receptor activity,” “toxic substance binding,” and “aminopeptidase activity”; GO:biological process (BP) category “protein-lipid complex subunit organization”; and GO:cellular component (CC) category “brush border” (Figure 1J). We found enriched expression of DPP4 (dipeptidyl peptidase 4) and ANPEP (alanyl aminopeptidase), which both encode for known receptors of human CoVs (Qi et al., 2020) and were similarly enriched in the lipoprotein-producing subcluster (Figure S1F). In particular, DPP4 is the receptor for MERS (Middle Eastern respiratory syndrome)-CoV, whereas ANPEP is a receptor for human coronavirus 229E, among other human CoVs (Qi et al., 2020). Interestingly, ChP cells of this subcluster also express lipoproteins that are also required for assembly of hepatitis C virus (HCV), as well as some HCV entry factors, such as SCARB1 and CD81 (Aizawa et al., 2015; Grassi et al., 2016; Wrensch et al., 2018; Figure S1G). Together, these data suggest that (1) more mature lipoprotein-expressing ChP cells express entry factors required for SARS-CoV-2 infection and (2) neuronal progenitors and neurons do not specifically express SARS-CoV-2 entry factors.

### SARS-CoV-2 Spike Pseudovirus Only Infects ChP Cells of Brain Organoids

To examine SARS-CoV-2 neurotropism, we incubated brain organoids with mixed identities, including ChP and cortical tissue with SARS-CoV-2 Spike pseudovirions carrying a 19-amino-acid deletion (c19) from the C terminus, which allows for better expression and integration of the spike into the lentivirus (Giroglou et al., 2004; Ou et al., 2020). SARS-CoV-2 Spike pseudovirions allow investigation of viral entry without other effects of live CoV, such as cell death or viral replication, and because they encoded for GFP, we could visualize infected cells only (Giroglou et al., 2004). As a negative control, pseudovirions lacking viral envelope glycoprotein (Δenv) were used for the infection. Vesicular stomatitis virus G protein (VSV-G) pseudotyped lentivirus (VSV) with broad viral tropism was used as a positive control (Figures S2A–S2C). A biochemical assay of the viral reverse transcriptase (RT) was used to assess viral particle production and subsequent titration in ACE2-overexpressing 293T cells (Figures S2A–S2C). We initially examined infection by direct observation of GFP fluorescence (Figure S2D), which clearly showed positive cells, but also some putative autofluorescence in the green channel. Indeed, we found that dead or dying cells displayed quite bright fluorescence in the same channel as GFP (Figure S2E). Therefore, to be sure we were observing true GFP signal from infected cells, we added a step of immunostaining with a GFP antibody, which allowed for accurate assignment of infected cells.

**Figure 2:**
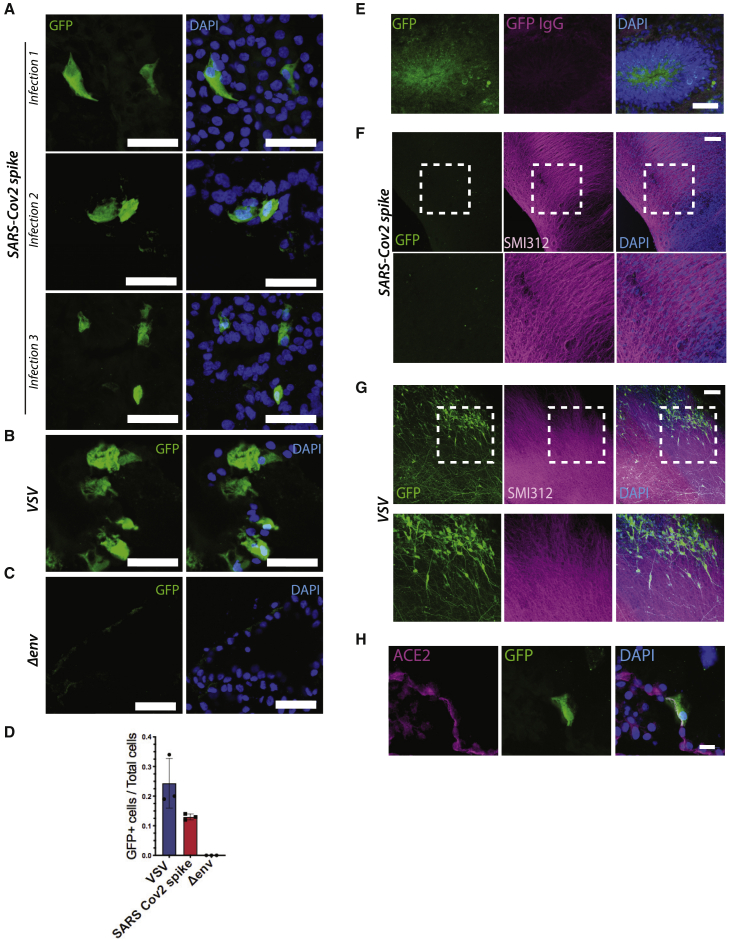
SARS-CoV-2 Spike Pseudovirus Infects ChP Cells, but Not Other Brain Cells, of Cerebral Organoids (A) Representative confocal images of ChP epithelial tissue from organoids-infected SARS-CoV-2 spike pseudovirions. GFP-positive cells, as detected by GFP antibody, are shown from three independent experiments with organoids aged 56 days (infection 1), 73 days (infection 2), and 78 days (infection 3). Nuclei in blue are stained with DAPI. Scale bar: 50 μm. (B) Representative confocal images of ChP epithelial tissue from organoids infected with VSV-G lentivirus. Scale bar: 50 μm. (C) Representative confocal images of ChP epithelial tissue from organoids infected with lentivirus pseudovirions lacking viral glycoprotein of the envelope (Δenv). Scale bar: 50 μm. (D) Quantification of mean GFP-positive cells over total counted cells for VSV-G, SARS-CoV-2 spike, and Δenv-lentiviral-infected ChP epithelial cells from organoids (n = 100 cells counted for each of the three independent experimental repeats, error bars are SD). (E) Representative confocal image of a cortical lobe of a day 78 cerebral organoid infected with SARS-CoV-2 pseudovirions showing an example of a false-positive signal due to GFP autofluorescence and stained with anti-GFP antibody (in magenta). Nuclei in blue are stained with DAPI. Scale bar: 50 μm. (F) Representative images of a day 78 ALI-CO infected with SARS-CoV-2 spike pseudovirions and immunostained with axonal marker SMI312 in magenta, anti-GFP antibody in green, and DAPI in blue. Scale bar: 100 μm. (G) Representative images of a day 78 ALI-CO infected with VSV lentivirus and immunostained with axonal marker SMI312 in magenta, anti-GFP antibody in green, and DAPI in blue. Scale bar: 100 μm. (H) Higher magnification image of ChP epithelial tissue from organoid immunostained for ACE2 in magenta, GFP, and DAPI. Scale bar: 20 μm.

We performed three independent infections with SARS-CoV-2 Spike pseudovirions of organoids aged more than 55 days to ensure a mature identity, which revealed viral tropism for ChP epithelial tissue, as indicated by the GFP antibody-positive signal (Figure 2A). Positive control for the infection with VSV lentivirus confirmed a broader tropism (Figures 2B and S2F), whereas no positive signal was detectable in organoids infected with Δenv lentivirus (Figures 2C and S2F). Quantification of the ratio of GFP-positive cells to total cells showed that SARS-CoV-2 infected around 13% of cells in the ChP epithelium (Figure 2D).

Interestingly, we found that neuronal regions of organoids with mixed identity did not seem to get infected with the virus (Figure S2G), and the only signal we could detect in the green channel was autofluorescence (Figures 2E and S2G). To further investigate the ability of SARS-CoV-2 Spike pseudovirus to infect cortical tissue and neurons, we infected air-liquid interphase cerebral organoids (ALI-COs) (Giandomenico et al., 2019), which are long-term cultures of organoids that lead to improved neuronal maturity and function. ALI-COs infected with SARS-CoV-2 and immunostained for SMI312 to visualize neurons showed no specific GFP-positive signal (Figure 2F) when compared to the signal detected in ALI-COs infected with the positive control (Figure 2G). This indicated the lack of signal upon SARS-CoV-2 Spike infection was not due to defective GFP expression but rather a lack of viral entry.

These findings support the hypothesis that, also in the brain, susceptibility to infection by SARS-CoV-2 is governed by expression of ACE2, which is only present on ChP epithelial cells of the organoids. Further supporting this claim, confocal imaging of infected cells immunostained for ACE2 showed positive signal on the membrane of SARS-CoV-2-infected ChP cells (Figure 2H).

**Figure 3:**
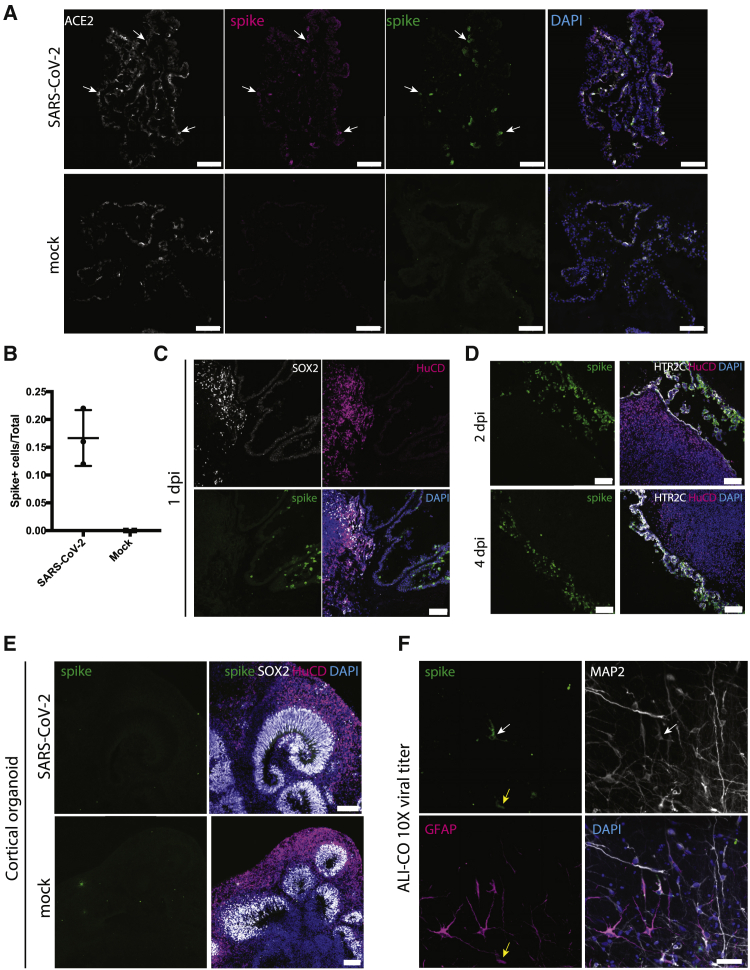
Live SARS-CoV-2 Specifically Infects ChP Epithelium (A) 1 day post-infection (dpi) of day 110 ChP organoids with either live SARS-CoV-2 or mock and staining for two independent antibodies (Abcam ab252690 spike glycoprotein in magenta and GeneTex GTX632604 in green) directed to the viral spike protein. Specific staining is only seen in the SARS-CoV-2 infection condition, with co-staining for ACE2 (arrows). Scale bars: 100 μm. (B) Quantification of infected cells staining positive for viral spike protein in ChP tissue infected with SARS-CoV-2 (n = 3 independent infections) compared with mock (n = 2 independent infections). Data are shown as mean with error bars representing SD. (C) Staining for viral spike protein in mixed identity day 117 telencephalic organoids at 1 dpi showing staining only in ChP tissue. Scale bar: 100 μm. (D) Staining for viral spike protein in day 124 mixed identity telencephalic organoids displaying adjacent cortical and ChP tissues at 2 dpi and 4 dpi. Scale bars: 100 μm. (E) Staining for viral spike protein in pure day 48 cortical organoids infected with either SARS-CoV-2 or mock showing no specific staining in either condition. Scale bars: 100 μm. (F) Staining for viral spike protein in day 156 ALI-COs infected for 2 days with 10 times the dose used for ChP or mixed-identity organoids. Note sparse staining of a neuron (white arrow) that is positive for MAP2 and a glial cell (yellow arrow) that is positive for GFAP. Scale bars: 50 μm.

Recent reports have similarly explored neurotropism of SARS-CoV-2 using brain organoids and neurospheres but with contradictory findings to those we observed here (Ramani et al., 2020; Song et al., 2020). One possible explanation is the use of pseudovirus versus live SARS-CoV-2, with other reports making use of live viral isolates. Therefore, to explore this possibility, we turned to infections with a live SARS-CoV-2 clinical isolate (Papa et al., 2020). We first infected mature ChP organoids with an equivalent amount of virus as we had performed using pseudovirions and observed highly comparable infection (Figures 3A and 3B). We next tested whether this same amount of virus was capable of infecting neurons and other cortical cells of telencephalic organoids with a mixed identity containing also ChP. We still found infection exclusively of the ChP in these conditions (Figure 3C), as also confirmed by co-staining for the ChP marker TTR (Figure S3A), suggesting that also live SARS-CoV-2 specifically infects the ChP.

We next sought to test whether there could be any conditions in which neurons or other CNS cell types may be infected with SARS-CoV-2. We did not observe any viral spreading from ChP to nearby cortical regions, even when in very close proximity and left for as long as 4 days (Figure 3D). However, one possibility is that the virus preferentially infects ChP, resulting in lower amounts of viral particles available to infect the surrounding neurons. We therefore tested infection with live virus on “pure” cortical organoids containing no ChP but still saw no specific staining for viral protein in neurons or neural progenitors (Figures 3E and S3B). Finally, we tested whether a combination of longer exposure to sliced organoids with internal neural regions exposed and much higher viral quantities would lead to infection. We performed infection of ALI-COs with 10 times the viral MOI used for ChP infection and were able to observe very sparse but specific neuronal and glial infection after 2 days post-infection (Figures 3F and S3C). This suggests that SARS-CoV-2 has a much lower infectivity of human neural cell types compared with ChP epithelium.

**Figure 4:**
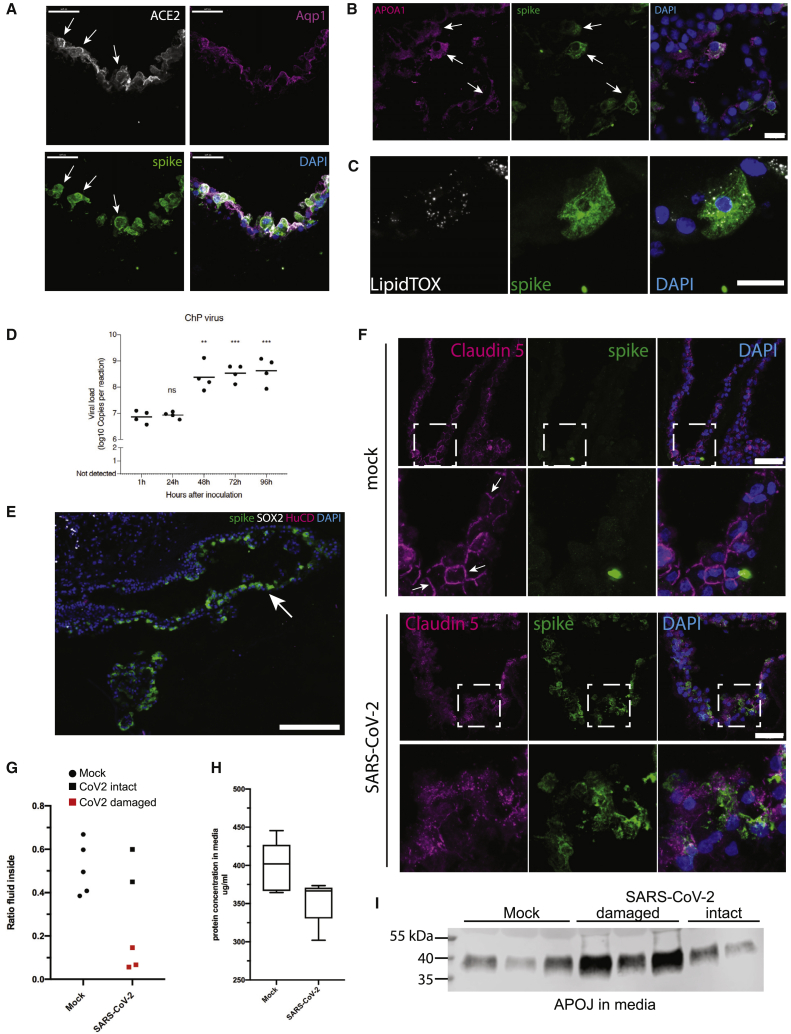
SARS-CoV-2 Disrupts ChP Epithelial Integrity and Barrier Function (A) Co-staining for ACE2 in infected cells of day 124 ChP tissue (arrows) with co-staining for the apical marker Aquaporin 1 (Aqp1). Scale bars: 50 μm. (B) Co-staining for APOA1 and viral spike protein in ChP epithelium after 1 dpi. Scale bar: 20 μm. (C) Co-staining for LipidTOX and viral spike in a ChP epithelial cell after 1 dpi. Scale bars: 20 μm. (D) qRT-PCR using primers and probes against the CDC N1 amplicon of SARS-CoV-2 in infected ChP organoids (days 117 and 124) over the course of 4 dpi. n = 4 organoids from 2 independent infections. ^∗∗^p = 0.002; ^∗∗∗^p < 0.001; two-tailed unpaired Student’s t test. (E) Staining for viral spike protein in a day 117 telencephalic organoid with intact CSF-like fluid-filled cysts (arrow) showing infection after application of virus on the basal (outer) surface. Scale bar: 200 μm. (F) Staining for tight-junction protein claudin 5 in day 117 ChP epithelium infected with SARS-CoV-2 or mock at 2 dpi. Note the presence of clearly demarcated junctions (arrows) in mock versus infection with SARS-CoV-2. Green fluorescent signal in mock represents typical nonspecific background, which does not label a cell. Scale bars: 50 μm. (G) Internal fluid volume as a ratio of the total excess volume in the final media and lysates collected at 4 dpi. Red data points are SARS-CoV-2-infected day 77 ChP organoids with damaged morphology as shown in Figure S4E. n = 5 organoids for each condition. (H) Total protein concentration in the media, as measured by Bradford assay, of organoids at 4 dpi with mock or SARS-CoV-2. n = 5 day 77 organoids for each condition. Whiskers are min and max. (I) Immunoblot for APOJ of media samples at 4 dpi of day 77 ChP organoids. Three mock samples were run alongside all five SARS-CoV-2-infected samples. Those labeled damaged refer to those organoids with damaged morphology (samples 1, 4, and 5) as shown in Figure S4E.

We next examined expression of ACE2 and markers of lipoprotein production to test whether these may explain the susceptibility of the ChP epithelium. Again, this infection matched ACE2 expression, with infected cells co-staining for the receptor (Figure 4A) in the ChP epithelium, whereas stromal cells of the ChP were uninfected (Figure S4A). We also observed expression of APOA1 and abundant lipid vesicles in cells of the ChP epithelium infected with SARS-CoV-2 (Figures 4B and 4C), whereas these cells were absent from cortical neuronal regions (Figure S4B).

We next examined whether ChP cells represent a permissive cell type for viral replication. We performed qRT-PCR for viral N1 and observed a significant increase in viral genome copies in organoid supernatant over the course of 4 days (Figures 4D and S4C), with the largest increase between 1 and 2 days of infection. This matched staining where we similarly observed larger numbers of cells infected in the ChP at 2 and 4 days post-infection (Figures 3C and 3D).

The ChP epithelium is highly polarized, with its basal side facing the vascularized stroma. Thus, potential viral entry *in vivo* would have to occur from the basal surface. However, we observed strongest ACE2 expression on the apical side, whereas there was much less present on basolateral surfaces (Figure 4A). To test whether this low level of ACE2 would nonetheless be sufficient to allow infection of ChP epithelium, we performed infections of intact tissues with clear, fluid-filled cysts, which we have previously demonstrated exhibit the same polarity as *in vivo* (Pellegrini et al., 2020). This experiment revealed abundant infection from the basal side (Figure 4E), suggesting that blood-borne virus would indeed be able to infect from the vascular compartment of the ChP.

We next examined the impact of SARS-CoV-2 infection on the function of the ChP epithelium, specifically its role as an integral part of the blood-CNS barrier. We observed a striking effect on tight junctions, labeled by claudin-5, as early as 2 days post-infection (Figure 4F), with increasing disruption by 4 days post-infection (Figure S4D), suggesting a potential breakdown of barrier integrity. To further test this, we observed ChP organoid morphology over time, which revealed striking damage to organoid integrity in 3 of 5 organoids at 4 days post-infection (and no change in morphology in all 5 mock-infected organoids; Figure S4E). A breakdown of barrier integrity would be expected to lead to leakage of the internal CSF-like fluid from within, which should be measurable as a decrease in volume of fluid within the organoid. Indeed, we observed a dramatic decrease in internal fluid in those organoids that also exhibited disrupted morphology (Figure 4G), indicating a complete breakdown of the barrier. Furthermore, because CSF has a much lower protein content than serum or media, we measured the protein concentration in the surrounding media and observed a reduction in the media of SARS-CoV-2-infected organoids, further indicating leakage and therefore dilution of media proteins (Figure 4H). Finally, we performed immunoblot of samples of the surrounding media for the CSF component APOJ and similarly detected leakage in those organoids with damaged morphology (Figure 4I). Taken together, these data suggest that SARS-CoV-2 is capable of infecting ChP epithelial cells, leading to a breakdown of barrier integrity.

## Discussion

Given the increasing reports of neurological symptoms associated with COVID-19 (Montalvan et al., 2020; Moriguchi et al., 2020), understanding viral tropism is of significant interest for the development of better treatments and for prevention of long-term adverse effects. Our findings indicate that SARS-CoV-2 does not readily infect neuronal cells but rather infects ChP epithelial cells of the brain. This finding is consistent with the high levels of expression of SARS-CoV-2 entry factors, such as ACE2 and TMPRSS2, in the ChP *in vivo* and *in vitro*, compared to other brain regions (Chen et al., 2020), as well as recent data from neural organoids showing minimal neuronal susceptibility to SARS-CoV2 and rather efficient ChP infection, leading to transcriptional deregulation and cell death (Jacob et al., 2020). Furthermore, our findings of susceptibility of lipoprotein-producing cells matches closely the susceptibility seen in other organs, such as the intestine (Lamers et al., 2020).

A likely interpretation of these results is that the neurological symptoms reported in COVID-19 patients are mainly due to an indirect, secondary consequence of viral infection of support cells in the brain, rather than neurons themselves. We show that infection of the ChP epithelium by SARS-CoV-2 leads to disruption of the B-CSF-B, which is in close agreement with recent clinical data demonstrating leakage of blood proteins into CSF in more than 40% of patients tested (Neumann et al., 2020). Although this could then allow entry and spread of the virus into the brain, our results suggest that neural cells are minimally susceptible, even when exposed to high quantities of virus. Furthermore, substantial SARS-CoV-2 within the brain and CSF does not seem to be a widely reported finding (Neumann et al., 2020; Schaller et al., 2020). Nonetheless, barrier breakdown could allow abnormal entry of immune cells and cytokines, leading to harmful neuroinflammation.

A prerequisite for infection of the ChP epithelium, or indeed other parts of the brain, would be presence of the virus in the circulating bloodstream. Given the fact that detection of SARS-CoV-2 in serum is uncommon (Puelles et al., 2020) and the primarily respiratory symptoms of COVID-19, this may also explain the relatively rare occurrence of severe neurological symptoms in patients. However, once present in the blood, the ChP would represent an easily accessible site within the brain due to its fenestrated and leaky capillaries. This exposure is likely why it is also a site of immune surveillance by macrophages, dendritic cells, and monocytes, and infection of ChP epithelial cells can lead to proinflammatory cytokine production and recruitment of T cells that can initiate neural tissue injury (Ransohoff and Engelhardt, 2012). Furthermore, the B-CSF-B might be a particularly vulnerable barrier to invasion of pathogens and immune cells because the tight junctions between cells have lower electrical resistance compared to the BBB (Redzic, 2011; Schwerk et al., 2015). Thus, the niche surrounding the B-CSF-B makes it a likely target for both viral infection and entry of inflammatory cytokines and immune cells.

It is interesting to note that, among the neurological disturbances seen in this and previous CoV outbreaks, chronic fatigue and nonrestorative sleep disturbances have been noted (Moldofsky and Patcai, 2011). Indeed, multiple viruses, such as HCV, HIV, and EBV (Epstein-Barr virus), have been linked to chronic fatigue syndrome (CFS), also known as myalgic encephalomyelitis (ME) (Payne et al., 2013; Rasa et al., 2018; Williams et al., 2019). Although the exact cause of CFS/ME is still obscure, abnormal levels of a number of cytokines, such as IL-10, have been observed in CSF from patients with CFS/ME (Natelson et al., 2005), suggesting an imbalance in neuroimmune modulation. In HCV in particular, the most common extrahepatic symptom in patients is chronic fatigue (Poynard et al., 2002), and key mediators of HCV assembly are apolipoproteins, with regulation of lipoprotein metabolism being important for the HCV replication cycle (Aizawa et al., 2015; Grassi et al., 2016). Lipoproteins are mainly synthesized by hepatocytes and intestinal cells (Aizawa et al., 2015); however, we also describe the expression of viral entry factors for HCV and for SARS-CoV-2 in mature lipoprotein-producing cells of the ChP.

Together, these observations suggest the intriguing possibility that the ChP epithelium is particularly susceptible to infection by viruses and that this susceptibility may lead to breakdown of the B-CSF-B and development of neurological complications, such as CFS/ME. In the case of SARS-CoV-2, this susceptibility seems to be dictated by the expression of ACE2, which is primarily expressed in mature lipoprotein-producing ChP epithelium. Thus, our findings suggest that the neurological symptoms are not due to a direct effect on neurons but rather a consequence of the damage to this important barrier, resulting in leakage and proinflammatory changes in the CSF.

### Limitations of Study

One limitation of this study is the lack of examination of *post mortem* ChP from COVID-19 patients. Obtaining these samples is notoriously difficult, and the collection of brain tissue from COVID-19 patients is complicated by safety concerns with regard to accessing the brain of a patient with a contagious pathogen (Hanley et al., 2020). A second limitation comes from further safety concerns when working in BSL3 conditions, in particular, the inability to use sharps. This limits our ability to selectively extract fluid from within ChP organoids without media contamination, making it impossible to specifically test for virus or proteins within the CSF compartment in a safe manner. Third, the lack of vasculature in the organoid might represent an additional limitation, but because the capillaries surrounding the ChP epithelium are fenestrated and the stromal tissue is cell sparse, the media compartment of these studies should model the environment surrounding ChP epithelium *in vivo*. Thus, if virus is present in the blood, it would be likely to reach the epithelial cells in a similar fashion to the experiments carried out in this study. Finally, although ChP organoids seem to reach quite an advanced stage of maturity, neurons within telencephalic or cortical organoids are not thought to reach such an advanced stage. Thus, although we show that these fetal-stage neuronal tissues do not appear to be susceptible to SARS-CoV-2 (matching the fact that no congenital brain malformations have so far been reported), it is possible that more mature, adult-staged neurons may be susceptible. This would require further investigation, and additional *post mortem* analyses would greatly improve our understanding.

## STAR★Methods

### Key Resources Table

**Table:** 

REAGENT or RESOURCE	SOURCE	IDENTIFIER
**Antibodies**		

sheep anti-TTR	Abcam	Cat# ab9015; RRID:AB_306943
rabbit anti-ACE2	Abcam	Cat# ab108209; RRID:AB_10862654
goat anti-ACE2	R&D Systems	Cat# AF933; RRID:AB_355722
rabbit anti-HTR2C	Abcam	Cat# 5315-1; RRID:AB_10898403
mouse anti-TMPRSS2	SantaCruz	Cat#Sc515727
rabbit anti-claudin 5	ThermoFisher	Cat# 34-1600; RRID:AB_86930
rabbit anti-Sox2	Abcam	Cat# ab97959; RRID:AB_2341193
rabbit anti-APOA1	ThermoFisher	Cat# PA5-88109; RRID:AB_2804657
sheep anti-Tbr2	R&D Systems	Cat# AF6166; RRID:AB_10569705
rat anti-CTIP2	Abcam	Cat# ab18465; RRID:AB_2064130
mouse anti-HuC/D	Life Technologies	Cat# A-21271; RRID:AB_221448
rabbit anti-DLK1	Abcam	Cat# ab21682; RRID:AB_731965
rabbit anti-GFAP	Abcam	Cat# ab7260; RRID:AB_305808
chicken anti-MAP2	Abcam	Cat# ab5392; RRID:AB_2138153
chicken anti-GFP	Abcam	Cat# ab13970; RRID:AB_300798)
goat anti-MSX1	R&D Systems	Cat# AF5045; RRID:AB_2148804
rabbit anti-ND2	Abcam	Cat# ab104430; RRID:AB_10975628
mouse anti-SMI312	BioLegend	Cat#837934
rabbit anti-Aqp1	Millipore	Cat# AB2219; RRID:AB_1163380
rabbit anti-clusterin (D7N2K)	Cell Signaling Technology	Cat# 34642; RRID:AB_2799057
mouse anti-SARS-CoV-2 spike glycoprotein	GeneTex	Cat# GTX632604; RRID:AB_2864418
AlexaFluor 488, 568, 647 donkey anti-rabbit IgG (H+L)	Life Technologies	Cat#A32790, A10042, A31573
AlexaFluor 488, 568, 647 donkey anti-mouse IgG (H+L)	Life Technologies	Cat#A21202, A10037, A31571
AlexaFluor 647 donkey anti-sheep IgG (H+L)	Life Technologies	Cat#A21448
AlexaFluor 488, 647 donkey anti-goat IgG (H+L)	Life Technologies	Cat#A11055, A21447
AlexaFluor 488 donkey anti-rat IgG (H+L)	Life Technologies	Cat#A21208
AlexaFluor 647 goat anti-mouse IgG2b	Life Technologies	Cat#A21242
HCS LipidTox	ThermoFisher	Cat#H34477

**Bacterial and Virus Strains**		

VSV-G and SARS Cov2 pseudotyped HIV-1 virions	(Papa et al., 2020)	doi: http://biorxiv.org/lookup/doi/10.1101/2020.08.13.243303
SARS-CoV-2/human/Liverpool/REMRQ0001/2020	Isolated by Lance Turtle (University of Liverpool), David Matthews and Andrew Davidson (University of Bristol)	N/A

**Chemicals, Peptides, and Recombinant Proteins**		

Matrigel	Corning	Cat#354234
CHIR99021	Tocris	Cat#4423
Recombinant human BMP4	R&D	Cat#314-BP
StemFlex culture media	GIBCO	Cat#A3349401
Rock Inhibitor Y27632	Merck	Cat#688000-5
Accutase	Sigma	Cat#A6964
DMEM F-12	Invitrogen	Cat#11330-032
Neurobasal Medium	Invitrogen	Cat#21103049
MEM-Non-Essential Amino Acids	Sigma	Cat#M7145
GlutaMAX	Invitrogen	Cat#35050-038
Penicillin-Streptomycin	Sigma	Cat#P0781
N2	Invitrogen	Cat#17502048
B27	Invitrogen	Cat#17504044
Geltrex LDEV-Free Reduced Growth Factor Basement Membrane Matrix	Life Technologies	Cat#A1413202
DAPI	Life Technologies	Cat#D1306
Prolong Diamond Antifade mountant	Invitrogen	P36965
β-Mercaptoethanol	Life Technologies	Cat#31350-010

**Critical Commercial Assays**		

Cerebral Organoid kit	Stem Cell Technologies	Cat#08570, 08571
QIAmp Viral RNA mini kit	QIAGEN	Cat#52904
Bio-Rad Protein Assay Kit I	Biorad	Cat#5000001
IDT 2019-nCoV RUO kit	IDT	Cat#10006770

**Deposited Data**		

Single-cell RNA-sequencing	(Pellegrini et al., 2020)	NCBI GEO: GSE150903
Single-cell RNA-sequencing	(Zeisel et al., 2018)	NCBI SRA: SRP135960
Single-cell RNA-sequencing	(Cao et al., 2019)	NCBI GEO: GSE119945

**Experimental Models: Cell Lines**		

HEK293T CRL-3216	ATCC	N/A
Vero cells	ATCC	N/A
H9 ES	WiCell	Cat#WA09

**Oligonucleotides**		

Fwd primer: AACATGCTCGAGGGCCTT	(Vermeire and Verhasselt, 2013)	N/A
Rev primer: GCCTTAGCAGTGCCCTGTCT	(Vermeire and Verhasselt, 2013)	N/A
Probe: TGGGATGCTCCTACATG	(Vermeire and Verhasselt, 2013)	N/A

**Recombinant DNA**		

Plasmid: pCRV-1 for HIV-1 Gag/Pol	(Naldini et al., 1996)	N/A
Plasmid: CSGW for GFP expression	(Zennou et al., 2004)	N/A
Lentiviral packaging plasmid pMDG2	Addgene	plasmid #12259
pCAGGS-Spike Δc19	(Papa et al., 2020)	doi: http://biorxiv.org/lookup/doi/10.1101/2020.08.13.243303

**Software and Algorithms**		

ImageJ	National Institute of Health	https://imagej.nih.gov/ij/
Prism	GraphPad	https://www.graphpad.com/
R Studio	R	https://rstudio.com/
Seurat v3.1.4	(Stuart et al., 2019)	https://satijalab.org/seurat/install.html

### Resource Availability

#### Lead Contact

Further information and requests for resources and reagents should be directed to and will be fulfilled by the Lead Contact, Madeline A. Lancaster (madeline.lancaster@mrc-lmb.cam.ac.uk).

#### Materials Availability

All data are available in the main text or the Supplemental Information. H9 cells are available from WiCell under a material transfer agreement with WiCell. Pseudovirions carrying SARS-CoV-2 spike, live SARS-CoV-2 isolate, and ACE2 overexpressing HEK293 cells have been previously published (Papa et al., 2020). These tools are available upon request. This study did not generate new unique reagents.

#### Data and Code Availability

The single cell data from Pellegrini et al. (2020) are available at http://chporg.cells.ucsc.edu and have been deposited on NCBI GEO (GSE150903). The data that support these findings are available from the Lead Contact, Madeline A. Lancaster (madeline.lancaster@mrc-lmb.cam.ac.uk) upon request.

### Experimental Model and Subject Details

#### Cells and plasmids

HEK293T CRL-3216 cells were purchased from ATCC and authenticated by the supplier. All cells were regularly tested and were mycoplasma free. HEK293T cells were maintained in Dulbecco’s modified Eagle’s medium (DMEM) with 10% FBS, 2 mM Lglutamine, 100 U/ml penicillin, and 100 μg/ml streptomycin (GIBCO) at 37**°**C with 5% CO2. Vero cells were purchased from ATCC and maintained in DMEM supplemented with 10% FBS, 100 U/ml penicillin and 100mg/ml streptomycin. Cells tested negative for mycoplasma before virus production and infection experiments. Vero-hACE2 were generated by transducing Vero cells with lentiviral particles expressing hACE2 ORF and cultured in DMEM 10% FCS with 5 μg/ml blasticidin. Vero-hACE2-TMPRSS2 were generated by transducing Vero-hACE2 with lentiviral particles expressing TMPRSS2 ORF and maintained in DMEM 10% FCS with addition of 5 μg/ml blasticidin and 1 μg/ml puromycin. H9 ES cells were obtained from WiCell (WA09) and have been approved for these studies by the UKSCB Steering Committee. Human ES cells were maintained in culture with StemFlex culture media (GIBCO A3349401) on growth factor reduced Matrigel coated dishes (Corning).

Vectors for viral production were: pCRV-1 for HIV-1 Gag/Pol (Naldini et al., 1996), and CSGW for GFP expression (Zennou et al., 2004). Lentiviral packaging plasmid pMDG2, which encodes VSV-G envelope, was used to pseudotype infectious virions (Addgene plasmid # 12259). pCAGGS-Spike Δc19 was generated from pCAGGS-Spike by digesting the vector backbone with EcoR1 and NheI and subsequently gel purified. Q5 polymerase (NEB) was used to amplify the spike protein fragment using the 3′ primer to make a C-terminal 19 amino acid deletion. Primers with a 15bp overlap with the vector backbone were used. After PCR, the fragments were treated with Dpn1 for 1h and subsequently gel purified. The fragment was then inserted into the vector using In-Fusion assembly (Takara Inc.). The plasmid was checked by sequencing.

For the generation of ACE2 overexpressing HEK293T cells, the human ACE2 ORF was PCR amplified from Addgene plasmid 1786 and C-terminally fused with the porcine teschovirus-1-derived P2A cleavage sequence (ATNFSLLKQAGDVEENPGP) followed by the blasticidin resistance gene. This continuous, single ORF expression cassette was transferred into pLenti6-Dest_Puro by gateway recombination. Lenti-viral particles were generated by co-transfection of HEK293T cells with pLenti6-Dest_Puro_ACE2-2A-Bla, pCMVR8.74 (Addgene plasmid 22036) and pMD2.G (Addgene plasmid 12259) using PEI. Supernatant containing virus particles was harvested after 48 h, 0.45 um filtered, and used to infect naive HEK293T cells. Transduced cells stably expressing ACE2 were selected with 5 ug/ml blasticidin.

#### Cerebral and ChP organoid culture conditions

Telencephalic cerebral organoids were prepared from single cell suspension of human ES cells as previously described (Lancaster and Knoblich, 2014) and cultured with Stem Cell Technologies Cerebral Organoid kit (catalog n. 08570, 08571). Briefly, EBs were generated by seeding 2000 cells in a 96-well U-bottom low attachment plate with EB media and 50μM Y-27632 ROCK inhibitor for 3 days. EB media was replaced on day 5 with NI media in the same 96 well plate. At day 7, EBs were embedded in 30μl Matrigel (Corning) using sheets of dimpled parafilm and incubated for 20min at 37°C as previously detailed (Lancaster and Knoblich, 2014). EBs were then transferred to a 6-well plate in 3ml of Expansion media per well. From day 30, dissolved Matrigel (1:50) was added to the Maturation media. ChP organoids were generated by adding a pulse treatment of BMP4 (20ng/ml) and CHIR (3μM) from day 10 to day 14 of the cerebral organoid protocol, as previously published (Pellegrini et al., 2020).

Air-Liquid Interface Cerebral Organoid (ALI-CO) culture was performed as previously described (Giandomenico et al., 2019). Briefly, mature organoids (day 55) were collected, washed in HBSS without Ca^2+^ and Mg2+ and embedded in 3% low-gelling-temperature agarose (Sigma, A9414) at 40°C in embedding molds (Sigma, E6032). Agarose blocks were then incubated for 10-15min on ice and sectioned using a vibratome (Leica VT100S vibrating microtome) in cold HBSS. Sections (300μm-thick) were collected onto Millicell CM culture inserts (Millipore, PICM0RG50) in 6-well plates and left at 37°C for 1h to equilibrate with serum-supplemented slice culture media (SSSCM). SSSCM was then replaced with serum-free slice culture media (SFSCM). ALI-CO cultures were fed with SFSCM daily.

### Method Details

#### Preparation of viruses

Replication deficient VSV-G or SARS Cov2 pseudotyped HIV-1 virions were produced in HEK293T cells by transfection with pMDG2 or pCAGGS-Spike Δc19, pCRV GagPol and CSGW as described previously (Price et al., 2014). Viral supernatants were filtered through a 0.45 μm membrane at 48 hours post-transfection and pelleted through a 20% sucrose cushion for 2hrs at 28K. Pelleted virions were drained and then resuspended in DMEM. Viral stocks were quantified by qRT-PCR as described previously (Vermeire and Verhasselt, 2013) with modifications. In brief, 5 μl of virus stock was mixed with 5 μl lysis buffer (0.25% Triton X-100, 50 mM KCl, 100 mM Tris-HCl (pH 7.4), 40% glycerol, 0.1 μl RNase Inhibitor). After incubation at room temp for 10 mins, 90 μL nuclease-free water was added. 2 μl of lysate was mixed with 5 μl TaqMan Fast Universal PCR Mix, 0.1 μl MS2 RNA, 0.05 μl RNase Inhibitor and 0.5μl MS2 primer/probe mix (7.5μM primers Fwd: AACATGCTCGAGGGCCTT, Rev: GCCTTAGCAGTGCCCTGTCT, 3.7μM probe, TGGGATGCTCCTACATG), in a final volume of 10μl.

For titer determination of pseudotyped viruses, 293T ACE2 cells were plated into 96 well plates at a density of 7.5x10^3^ cells per well and allowed to attach overnight. Viral stocks were titrated in triplicate by addition of virus onto cells. Infection was measured through GFP expression measured by visualization on an Incucyte Live cell imaging system (Sartorius). Infection was enumerated as GFP positive cell area.

SARS-CoV-2 virus, named “SARS-CoV-2/human/Liverpool/REMRQ0001/2020,” used in this study was isolated by Lance Turtle (University of Liverpool), David Matthews and Andrew Davidson (University of Bristol). SARS-CoV-2 stock was prepared in Vero hACE2-TMPRSS2 cells by infecting monolayer of cells with 0.01 MOI of virus. Virus stock was harvested after 3 days by three freeze-thaw cycles and 5 min 300xg spin to remove cell debris. Virus titers were assessed by plaque assays in Vero ACE2/TMPRSS2 cells. For plaque assays, Vero hAce2-TMPRSS2 cells were seeded on 12-well dishes day prior infection. Next day serial dilutions of the supernatant (−1 to −6) were prepared and used to infect cells for 1h and then overlayed with 0.05% agarose in 2% FBS DMEM. After 3 days monolayers were fixed with 4% formaldehyde and the plaques were revealed with 0.1% toluidine blue staining.

#### Single-cell RNA-seq and *in vivo* expression analyses

scRNA-seq data was previously published (Pellegrini et al., 2020) and was analyzed using the Seurat v3 R package. The already normalized and scaled matrix was visualized by UMAP dimensionality reduction. Subclustering of ChP cell types was performed by first extracting and combining cells of the ChP mature epithelial, immature/hem, and stromal clusters, followed by FindNeighbors, FindClusters and UMAP dimensionality reduction on PCs 1-12 (as determined by ElbowPlot). For expression correlation analysis, pearson correlation was performed between ACE2 expression in all ChP cells and all other genes. The top 20 correlated genes were used for GO term enrichment analysis using gProfiler (Reimand et al., 2011).

*In vivo* human data were obtained from the Allen Human Brain Atlas (http://human.brain-map.org/). Z-scaled data were downloaded for the two ACE2 probes available for all samples and brain regions. Mean Z-score was calculated for all samples within each brain region for each probe.

*In vivo* mouse single cell data was obtained from http://cells.ucsc.edu. For adult mouse ChP, the expression matrix from the Mouse Nervous System single cell dataset that was previously published (Zeisel et al., 2018) was loaded into R and all cells with Ttr values greater than 2 were extracted and taken forward for analysis. For embryonic mouse ChP, cells with Ttr greater than or equal to 1 and no Alb were extracted and taken forward for analysis. These two datasets were then merged and analyzed using the Seurat v3 R package. Data were then normalized for read depth across cells, scaled, and underwent variable feature finding using SCTransform. Unbiased clustering was performed by principle component analysis (PCA) using ElbowPlot to guide selection of the number of dimensions (4 principal components), followed by FindNeighbors, FindClusters, and UMAP dimensionality reduction visualization.

#### Organoid infection experiments

Organoid infections were performed by addition of virus to the culture medium at an expected MOI of 0.5 for each virus based on viral titer determined in 293T ACE2 cells (in the case of pseudotyped virus) or plaque assay (in the case of live SARS-CoV-2). To calculate an MOI, counts from previous single cell dissociations (Pellegrini et al., 2020) were used to estimate cell numbers in organoids. Thus, 7x10^3^ PFU virus was added to ChP tissues with an estimated number of 17,000 cells to achieve MOI 0.5. For mixed identity or pure cortical organoids, with an increased cell number estimated at approximately 35,000 cells, we used 1.4x10^4^ PFU virus, or 1.4x10^5^ in experiments using 10-fold increased viral quantity. In the case of organoid ChP tissue, ChP epithelium was physically separated from the organoid and broken into three large pieces before infection, and SARS-CoV-2 virus or pseudovirions, negative control, or positive control was added to each of the three pieces. This provided an added internal control so that the experiment and control conditions were tested on tissues obtained from the exact same organoid.

#### qRT-PCR analysis of viral replication

RNA extractions from 140μl of inoculated culture medium were performed using the QIAGEN QIAmp Viral RNA mini kit and eluted in 80μl of AVE elution buffer. 5μl of extract were used per 20μl RT-qPCR reaction. Reaction mixes were set up according to the manufacturer’s instructions with the addition of a CDC-N1 SARS-CoV-2 probe/primer mix (IDT 2019-nCoV RUO kit) with a final concentration of 500nM for each primer and 125nM of the hydrolysis probe. One-step RT-qPCR reactions were run on a Viia7s real-time qPCR instrument with the following parameters: 1. 55°C for 10 minutes for reverse transcription 2. 95°C for 1 minute for enzyme activation. 3. 45 cycles of 10 s denaturation at 95°C and 30 s annealing/extension at 55°C. Fluorescence was recorded at the end of the extension step. To estimate viral copies per reaction, a dilution series of nCoV-2019 N-gene positive control (IDT) was performed in technical duplicates. Sample Cq values were then converted to viral copies per reaction using the standard curve’s linear regression model. Values in dot plots represent log10 transformed copies per reaction of individual biological samples over time. For samples where no virus was detected, a pseudo value of 0 was assigned to represent the data point on the graph. Raw data is available upon request.

#### Plaque assay

Monolayers of Vero ACE2 TMPRSS2 cells were infected with ten-fold serial dilutions of supernatant from infected cells as previously described (Papa et al., 2020) to determine viral titers for organoid infections. After 1 hour of infection, cells were overlayed with 0.05% agarose in culture media and incubated for 3 days. Cells were then fixed and stained with 0.1% toludine blue to visualize the plaques.

#### Immunostaining and confocal imaging

Organoids were fixed in 4% PFA overnight at 4°C and then moved to 30% sucrose buffer at 4°C for at least 24h. Organoids were then embedded in gelatin and sectioned as previously described (Lancaster and Knoblich, 2014). After blocking and permeabilisation with 0.25% Triton and 1% donkey serum buffer, sections were incubated overnight with primary antibodies according to their optimized instructions. To mark the nuclei, Dapi (1:1000) was used added with the secondary antibody incubation. Secondary antibodies labeled with Alexa 488, 568 and 647 were applied 1h at room temperature. Incubation with HCS LipidTox (1:1000 in PBS, ThermoFisher, H34477) was carrier for 20min after secondary antibody to visualize lipid droplets. Slides were then washed in PBS and mounted using Prolong Diamond mounting media. All the staining steps for ALI-COs were carried out in permeabilisation buffer and their duration was extended as previously described (Giandomenico et al., 2019).

Images were acquired using a Zeiss LSM 780 confocal microscope (Carl Zeiss) and prepared using Fiji (NIH). The Fiji Huttner plugin was used for the quantification of GFP-positive cells over total cells: 100 Dapi-stained nuclei were counted in each experiment and number of GFP-positive cells over total cells counted was then recorded.

#### Immunoblotting

For immunoblotting, organoids were snap-frozen in liquid nitrogen and homogenized in RIPA buffer with protease inhibitors (Roche) to produce total protein lysate. Protein samples were prepared using NuPAGE LDS Sample Buffer 4x and DTT 1M and heated at 95°C for 15min. Protein samples and ladder were loaded into a polyacrylamide gel and run for 2h at 100mV. Wet transfer in PDVF membrane (Immobilion) was carried out for 3h or overnight at 4°C. Membranes were blocked in 5% milk in PBS-T and incubated overnight at 4°C with primary antibodies (rabbit anti-ACE2, rabbit anti-Clusterin) and mouse anti-β-actin). After 3 washes in PBS-T, secondary antibodies Alexafluor conjugated were added for 1h at room temperature and membranes were images using a Li-COR Odyssey CLx Infrared Imaging System.

#### Barrier assays

For measurements of internal and external volumes, organoids were carefully handled throughout the experiment to avoid accidental barrier breakage. On day 4 post-infection, media was carefully removed and its volume measured. Organoids were then lysed in RIPA buffer and total lysate volume was measured. Excess volume in the media and in the lysates beyond the volumes of media and RIPA added were then calculated, and excess internal volume was reported as a ratio of the total excess volume.

For measurements of leakage into the media, Bradford assay was performed using Bio-Rad Protein Assay Kit (Cat#5000001) to measure total protein content of the media. Western blot for APOJ on equal volumes of media samples was performed as described above.

### Quantification and Statistical Analysis

Data were reported as the mean ± standard deviation, except where indicated in the figure legends, using a significant level of p < 0.05. The number of replicates is indicated in figure legends and “n” denotes the number of independent experiments. For statistical comparisons, data were analyzed by Student’s t test, comparing mock and infected. Cells infected for SARS-CoV-2 were manually counted over total DAPI+ cells using the Cell Counter of ImageJ (NIH). No statistical methods were used to pre-determine sample size.

## Supplemental Information

Document S1. Figures S1–S4

Document S2. Article plus Supplemental Information
