# Supplementary material for: SARS-CoV-2 Infects the Brain Choroid Plexus and Disrupts the Blood-CSF Barrier in Human Brain Organoids
Source: Cell Stem Cell. 2020 Dec 3;27(6):951–961.e5. doi: 10.1016/j.stem.2020.10.001 (PMC7553118; doi:10.1016/j.stem.2020.10.001)
Supplement: Document S1. Figures S1–S4 [file mmc1.pdf]

Cell Stem Cell, Volume *27*

Supplemental Information

SARS-CoV-2 Infects the Brain Choroid Plexus and Disrupts the Blood-CSF Barrier in Human Brain Organoids

Laura Pellegrini, Anna Albecka, Donna L. Mallery, Max J. Kellner, David Paul, Andrew P. Carter, Leo C. James, and Madeline A. Lancaster

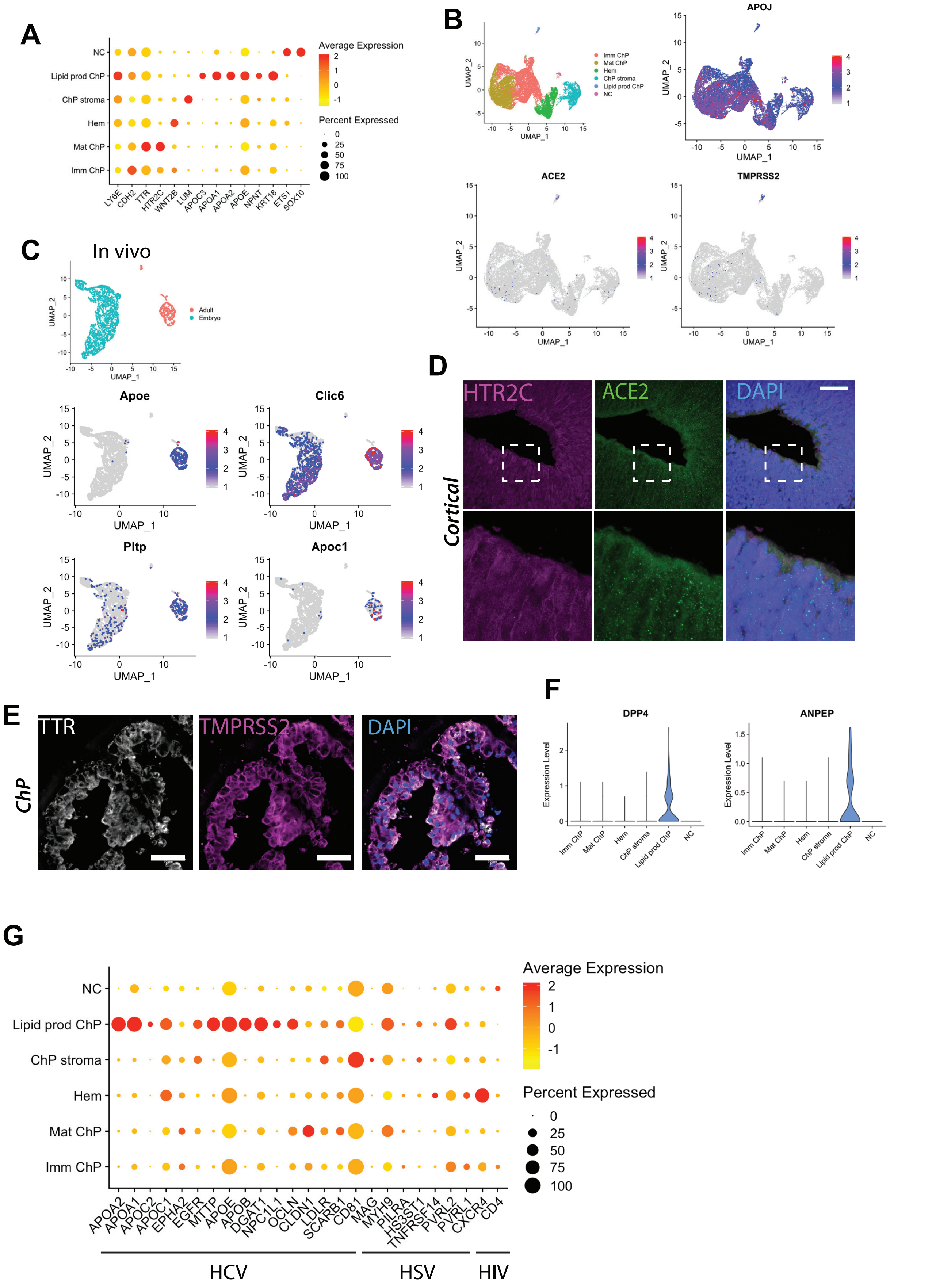

Figure S1, *related to Fig. 1*. Expression of apolipoproteins and viral entry factors in mature ChP cells.

(A) Dot plot showing average expression and percentage of cells expressing top genes in the six main ChP clusters identified by subclustering data from organoid scRNA-seq (Pellegrini et al., 2020). Lipid-producing ChP cells express apolipoprotein genes such as APOC3, APOA1, APOA2 and APOE as well as epithelial markers such as KRT18 (keratin 18) and lateral ventricle ChP marker LY6E (lymphocyte antigen 6E).
(B) Top left, UMAP showing subclusters of ChP; top right and bottom, feature plots for APOJ, ACE2 and the co-entry factor TMPRSS2, showing highest expression in the lipoprotein-producing cluster, as well as cells in other maturing ChP clusters that also express some lipoproteins.
(C) Top, UMAP colored according to input dataset: adult or embryonic mouse ChP cells. Bottom, feature plots for lipid-associated proteins Apoe, Pltp, Apoc1 as well as the ChP marker Clic6.
(D) Representative confocal images of cortical lobe from a day 73 telencephalic organoid immunostained for ChP marker HTR2C (magenta), ACE2 (green) and DAPI (blue). Scale bar: 50μm.
(E) Representative confocal images of a day 73 ChP epithelial region of a mixed identity organoid stained for ChP marker TTR (grey), TMPRSS2 (magenta) and DAPI (blue). Scale bar: 50μm.
(F) Violin plots showing expression levels of human coronavirus entry factors DPP4 and ANPEP in the lipid-producing ChP cell subcluster identified by scRNA-seq.
(G) Dot plot showing average expression and percentage of cells expressing HCV viral entry factors in the six subclusters identified by scRNA-seq of ChP organoids. ChP lipid-producing cells show the highest expression of HCV viral entry factors as compared to the other subpopulations.

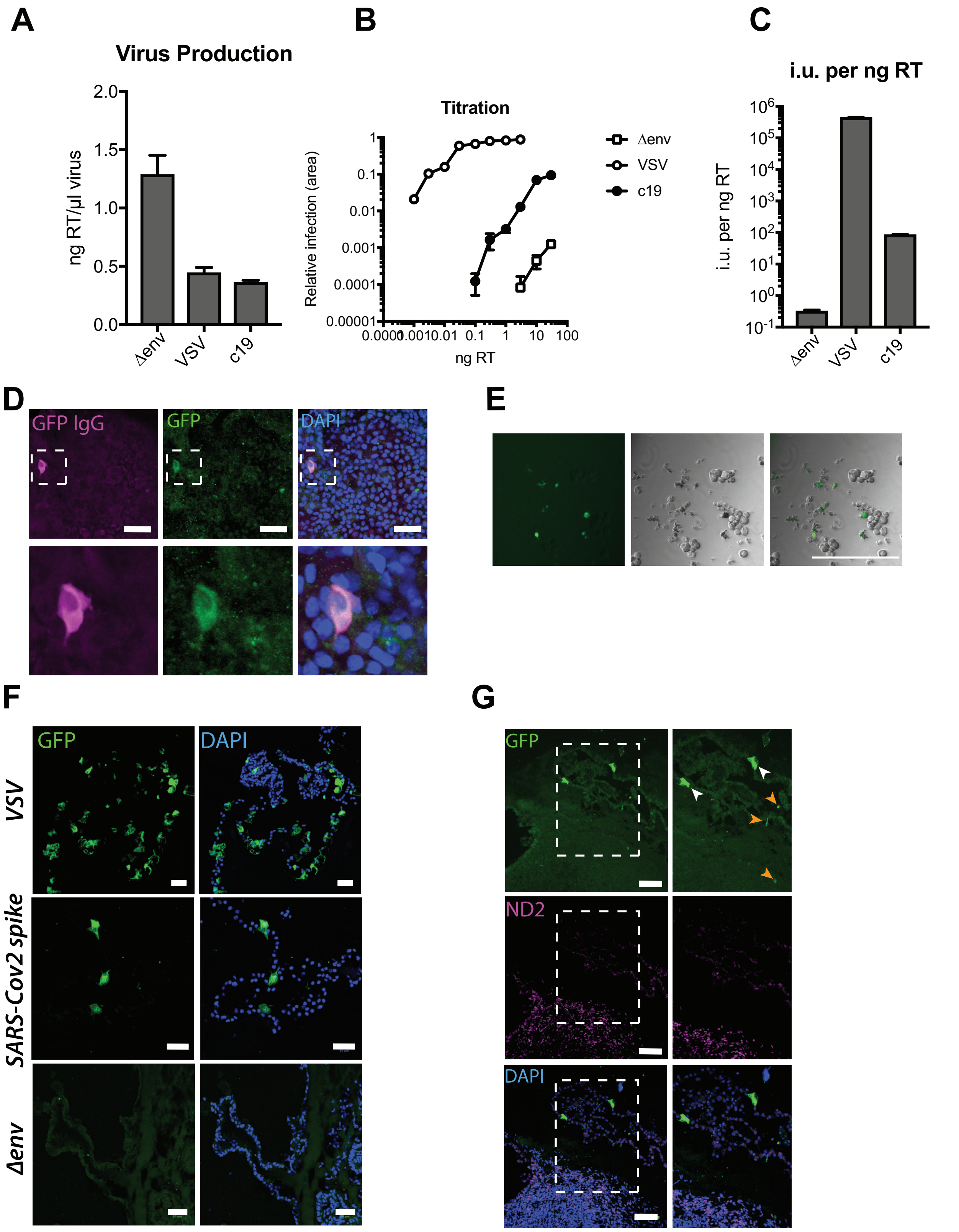

Figure S2, *related to Fig. 2.* Characterization of SARS-CoV-2 spike and control pseudoviruses.
(A) Quantification of viral particle production showing levels of RT enzyme per µl of virus for pseudovirions lacking viral glycoprotein of the envelope (Δenv), VSV lentivirus and SARS-CoV-2 spike with deletion of 19 aminoacids from the C-terminus (c19).

(B) Titration of Δenv, VSV and SARS-CoV-2 pseudoviruses onto ACE2 overexpressing 293T cells showing amount of virus added as quantity of RT (ng) on the x-axis.
(C) Quantification of infectious units equalised per particle addition (ng RT).
(D) Confocal images showing a ChP cell infected with SARS-CoV-2 spike pseudovirus immunostained with GFP antibody (GFP IgG, in magenta) and DAPI (blue). Scale bar: 50μm.
(E) Epifluorescence images of uninfected, dissociated ChP cells showing some autofluorescent dead cells. Scale bar: 200μm.
(F) Representative lower magnification confocal images of ChP tissue infected with VSV, SARS-CoV-2 spike and Δenv pseudovirions immunostained for GFP antibody and DAPI in blue. Scale bar: 50μm.
(G) Confocal images of an organoid region with cortical and ChP tissue immunostained for neuronal marker ND2 (magenta), GFP and DAPI (blue). White arrowheads indicate GFP-positive cells, orange arrowheads point to autofluorescence possibly derived from cellular debris.

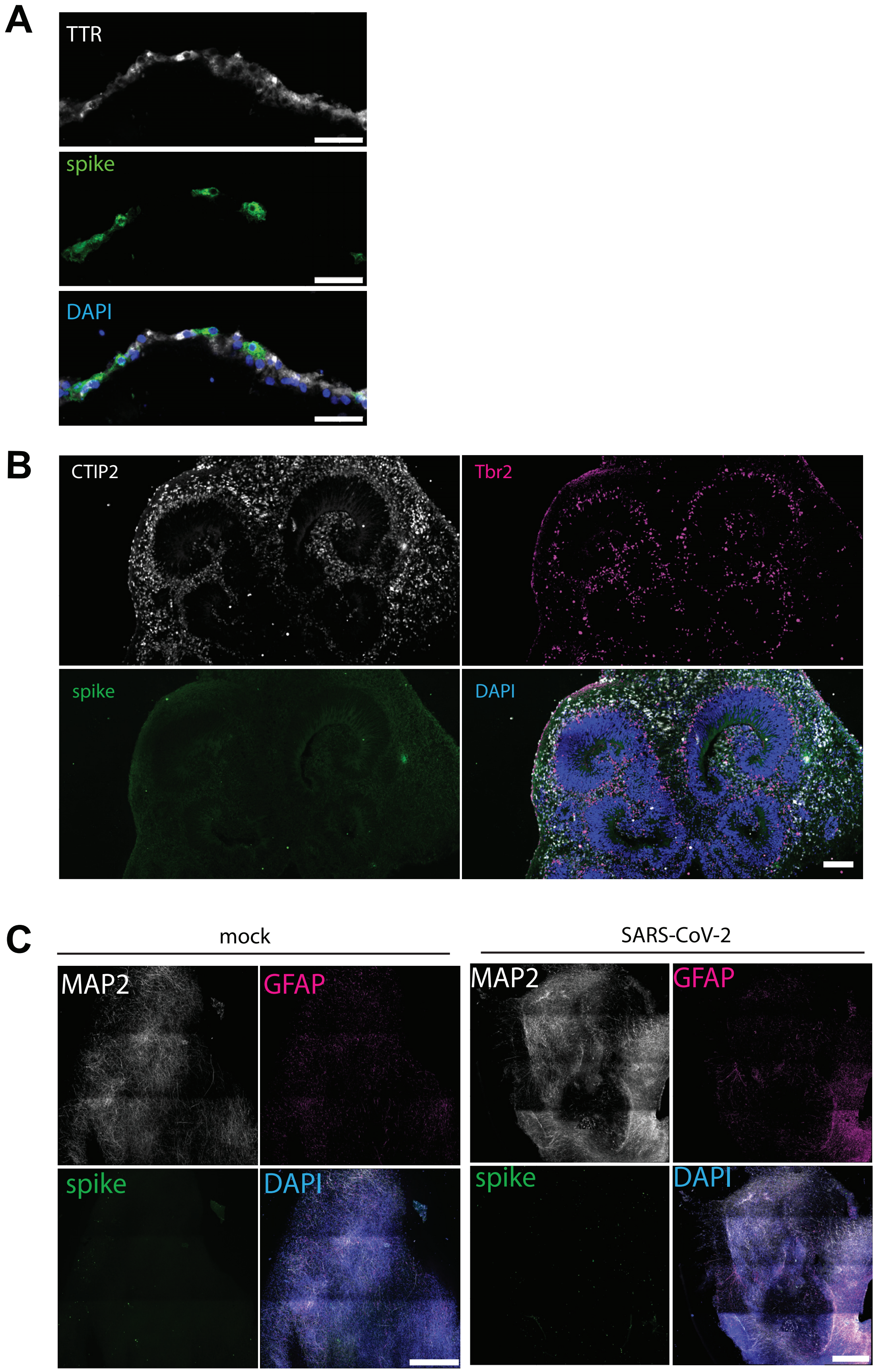

Figure S3, ***related to Fig. 3*****. Limited infection of neuronal tissue with live SARS-CoV-2.**

(A) Staining for SARS-CoV-2 spike protein within a region of infected ChP, recognizable by its morphology and co-staining for the marker TTR, at 1 day post-infection. Scale bar: 50µm.
(B) Staining for viral spike protein in a cortical organoid infected with live SARS-CoV-2 at 1 day post-infection showing no specific staining in CTIP2+ neurons or in TBR2+ intermediate progenitors. Scale bar: 100 µm.
(C) Overview image of ALI-CO infected with 10 times viral titer at 2 days post-infection showing only very sparse staining, despite abundant neurons (MAP2) and glia (GFAP). Scale bars: 500 µm.

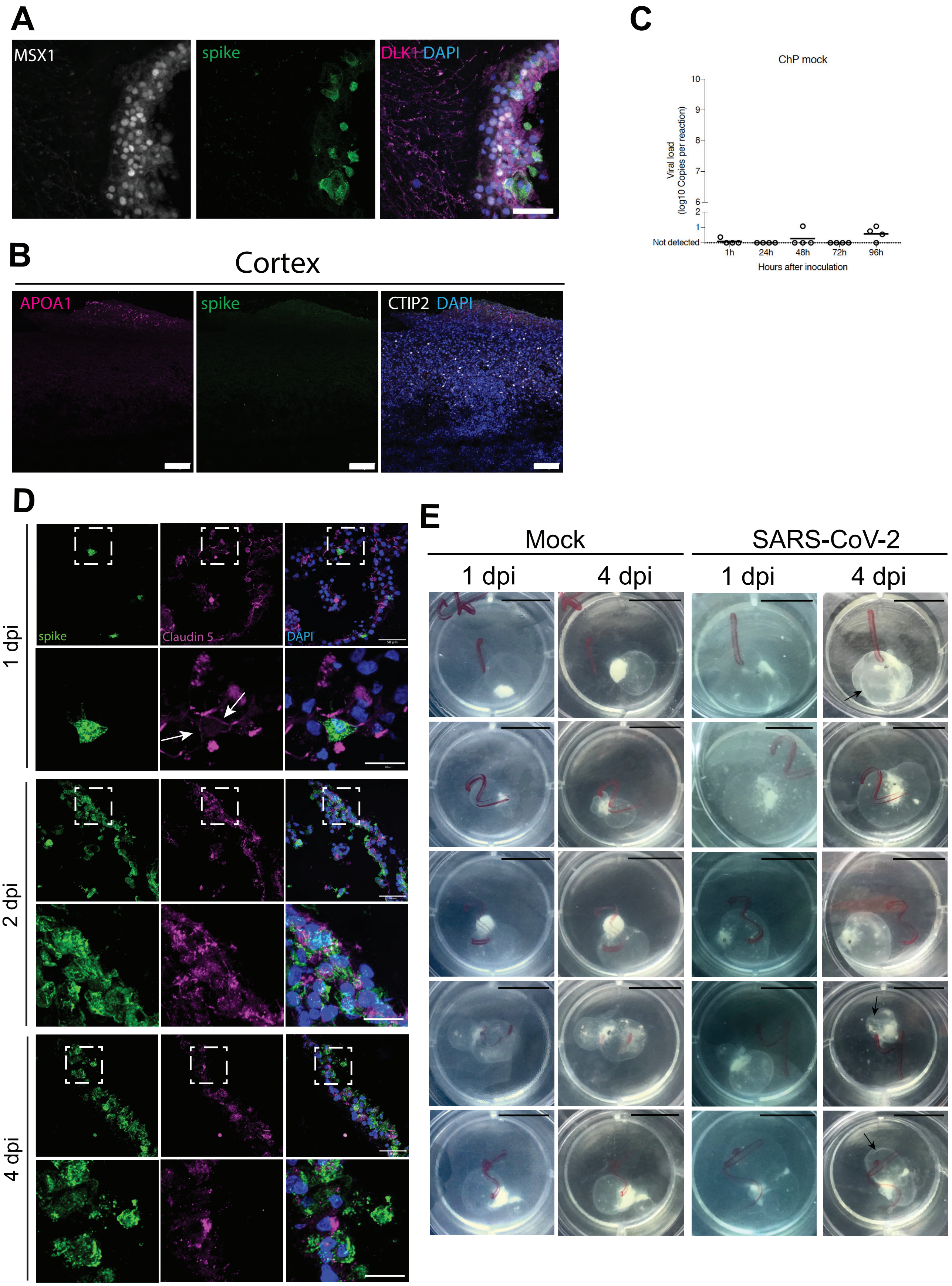

Figure S4, *related to Fig. 4*. Live SARS-CoV-2 productively infects the ChP and disrupts barrier integrity.

(A) Staining for viral spike protein and a marker of ChP stroma (DLK1) as well as ChP marker MSX1 showing no infection of stromal cells despite abundant infection of epithelium. Scale bar: 50 μm.
(B) APOA1 staining in infected cortical tissue showing no viral spike staining and only sparse APOA1 outside the cortical neuronal region marked by CTIP2. Scale bars: 100 μm.
(C) RT-qPCR using primers and probes against the CDC N1 amplicon of SARS-CoV-2 in mock infected ChP organoids over the course of 4 days post-infection. n=4 organoids from 2 independent infections.
(D) Staining for tight-junction protein Claudin 5 in 17-week-old ChP epithelium infected with SARS-CoV-2 at 1, 2, and 4 days post-infection (dpi). Note the presence of junctions (arrows) at 1 dpi but the lack of clear junctions at 2 and 4 dpi. Scale bars: 50 μm
(E) Bright field images of ChP organoids showing large fluid-filled cysts infected with SARS-CoV-2 and imaged at 1 and 4 days post-infection (dpi) compared with mock. Note the change in morphology in infected organoids 1, 4, and 5 which appear to have decreased tension as indicated by folds in the cyst membrane (arrows). Scale bars: 1 cm.
